# Coal dust alters β-naphthoflavone-induced aryl hydrocarbon receptor nuclear translocation in alveolar type II cells

**DOI:** 10.1186/1743-8977-6-21

**Published:** 2009-08-03

**Authors:** Mohamed M Ghanem, Lori A Battelli, Brandon F Law, Vincent Castranova, Michael L Kashon, Joginder Nath, Ann F Hubbs

**Affiliations:** 1 Genetics and Developmental Biology Program, West Virginia University, Morgantown, WV 26506, USA; 2 National Institute for Occupational Safety and Health, Center for Disease Control and Prevention, Morgantown, WV 26505, USA; 3 Dept. Animal Medicine, Faculty of Veterinary Medicine at Moshtohor, Benha University, 13736 Egypt

## Abstract

**Background:**

Many polycyclic aromatic hydrocarbons (PAHs) can cause DNA adducts and initiate carcinogenesis. Mixed exposures to coal dust (CD) and PAHs are common in occupational settings. In the CD and PAH-exposed lung, CD increases apoptosis and causes alveolar type II (AT-II) cell hyperplasia but reduces CYP1A1 induction. Inflammation, but not apoptosis, appears etiologically associated with reduced CYP1A1 induction in this mixed exposure model. Many AT-II cells in the CD-exposed lungs have no detectable CYP1A1 induction after PAH exposure. Although AT-II cells are a small subfraction of lung cells, they are believed to be a potential progenitor cell for some lung cancers. Because CYP1A1 is induced via ligand-mediated nuclear translocation of the aryl hydrocarbon receptor (AhR), we investigated the effect of CD on PAH-induced nuclear translocation of AhR in AT-II cells isolated from *in vivo*-exposed rats. Rats received CD or vehicle (saline) by intratracheal (IT) instillation. Three days before sacrifice, half of the rats in each group started daily intraperitoneal injections of the PAH, β-naphthoflavone (BNF).

**Results:**

Fourteen days after IT CD exposure and 1 day after the last intraperitoneal BNF injection, AhR immunofluorescence indicated that proportional AhR nuclear expression and the percentage of cells with nuclear AhR were significantly increased in rats receiving IT saline and BNF injections compared to vehicle controls. However, in CD-exposed rats, BNF did not significantly alter the nuclear localization or cytosolic expression of AhR compared to rats receiving CD and oil.

**Conclusion:**

Our findings suggest that during particle and PAH mixed exposures, CD alters the BNF-induced nuclear translocation of AhR in AT-II cells. This provides an explanation for the modification of CYP1A1 induction in these cells. Thus, this study suggests that mechanisms for reduced PAH-induced CYP1A1 activity in the CD exposed lung include not only the effects of inflammation on the lung as a whole, but also reduced PAH-associated nuclear translocation of AhR in an expanded population of AT-II cells.

## Background

Studying mixed exposure to polycyclic aromatic hydrocarbons (PAHs) and foreign particles, such as coal dust (CD) is extremely important in occupational health. Exposure to CD is associated with lung scarring and inflammation [1]. However, the impact of CD exposure on lung cancer in miners is difficult to interpret because most of them are smokers and consequently have exposure to carcinogenic PAHs in the cigarette smoke, which would normally induce cytochrome P4501A1 (CYP1A1) activity. Increased pulmonary CYP1A1 activity is associated with increased DNA adducts from PAH exposure and elevated risk of lung cancer [2]. Therefore, if inhalation of coal dust changes PAH-induced CYP1A1 activity, this could modify lung cancer risk. Establishing whether coal dust is a modifier of PAH metabolism and carcinogenesis is important for designing and interpreting epidemiology studies of lung cancer in coal miners.

CD does change PAH-induced CYP1A1 activity in a rodent model. In models of mixed exposure to respirable CD and PAHs, CD suppressed PAH-mediated cytochrome P4501A1 (CYP1A1) induction in rats [3]. Using the model PAH, β-naphthoflavone (BNF), our laboratory demonstrated decreased BNF-induced CYP1A1 induction in the rat lung 2 weeks after CD exposure [3,4]. The decrease in PAH-induced CYP1A1 expression in the CD-exposed lung was demonstrated using western blots from microsomes isolated from whole lung digests [3,4]. In addition, decreased CYP1A1 induction was demonstrated in alveolar septa in histologic sections of lung using immunofluorescence [3,4]. In histologic sections, the alveoli near the bronchioloalveolar junction demonstrated the greatest decrease in PAH-induced CYP1A1 expression [3]. Because alveolar type II (AT-II) cells strongly express cytokeratins 8 and 18 [5], AT-II cell hyperplasia was demonstrated by morphometric measurement of cytokeratin 8/18 immunofluorescence. In addition, co-localization of CYP1A1 and cytokeratin 8/18 expression in alveoli allowed detection of CYP1A1 expression in intact AT-II cells within the lung [3,4], and demonstrated decreased PAH-induced CYP1A1 in AT-II cells. Thus, the CD-exposed lung contains an expanded population of AT-II cells that are refractory to PAH-induced CYP1A1.

CYP1A1 is induced through activation of the aryl hydrocarbon receptor (AhR). Mature AhR is usually located in the cytoplasm complexed with chaperones including: heat shock protein 90, AhR-interacting protein [6], and P23 [7]. Prototypical ligands for AhR are the substrates for CYP1A1 and include PAHs, such as benzo(a)pyrene [8], BNF and 2,3,7,8,-tetrachlorodibezo-p-dioxin (TCDD) [9-11]. After ligand binding, AhR is released from the associated cytoplasmic proteins and the AhR nuclear localization signal is exposed [11]. This causes translocation of AhR to the nucleus where it dimerizes with the AhR-nuclear translocator (Arnt), forming a heterodimer complex containing the bound substrate. The AhR/Arnt heterodimer with bound substrate binds to the xenobiotic responsive element on the CYP1A1 enhancer, overcoming the repressive effect of nucleosome and initiating the CYP1A1 transcriptional process [12]. Therefore, the PAH-induced translocation of AhR-substrate complex to the nucleus is a key mechanism by which the CYP1A1 induction is initiated. Accordingly, investigating the translocation of AhR to the nucleus is extremely important in exploring the mechanism of CD-mediated suppression of CYP1A1 induction [3]. Because AT-II cells are a specific cell type demonstrating CD-mediated suppression of PAH-induced CYP1A1 [3,4], in this study, we investigated the hypothesis that AT-II cells from CD-exposed rats have decreased AhR translocation to the nucleus following *in vivo *PAH exposure.

## Methods

### Animals

Male viral antigen negative, Sprague-Dawley (Hla:(SD)CVF) rats (220–270 g BW at exposure time) were purchased from Hilltop Labs (Scottdale, PA). Rats were kept in an AAALAC-approved barrier animal facility at NIOSH, where food and water were supplied *ad libitum*. Rats were housed in ventilated shoebox cages on autoclaved hardwood (Beta-Chip; Northeastern Products, Inc., Warrensburg, NY) and cellulose bedding (ALPHA-dri; Shepherd, Watertown, TN) in HEPA-filtered, ventilated cage racks (Thoren Caging System, Inc., Hazleton, PA). The rats were acclimatized for one week before starting the experiment. The experimental protocol was reviewed and approved by the Institutional Animal Care and Use Committee.

### Experimental Design

The experimental design was based upon 1) the lung burden of 10–25 mg CD/g lung wet weight identified in coal miners with coal workers pneumoconiosis [13,14], and 2) a previous study showing that within this approximate range in the rat, AT-II cell hyperplasia was greatest at 40 mg/rat which is ~26.7 mg/g lung wet weight (the approximate lung wet weight for rats is 1.5 g). Sixteen male rats were randomized into 4 equal groups using a research randomizer program . Rats were intratracheally instilled with sterile saline or CD particles (CD; 40 mg/rat, ~160 mg/kg BW) in saline. Eleven days later, rats were injected intraperitoneally with BNF (50 mg/kg BW) suspended in filtered corn oil to induce CYP1A1. The BNF was injected daily for 3 days. Corn oil was injected as the control. Twenty-four hours after the last BNF or corn oil injection, rats were sacrificed and AT-II cells were isolated by digesting lung tissue with elastase as described later.

### CD characterization

Respirable CD was prepared from coal acquired at the Pittsburgh coal seam. The CD was characterized and fractionated to produce respirable CD particles as previously described [3,15]. These particles were less than 5 microns in diameter, had a mass aerodynamic diameter of 3.4 microns and a surface area of 7.4 m^2^/g. Numerically, silica particles comprised 2.3% of the total particle number. The particles contained 0.34% total iron [3,15]. The CD particles were additionally characterized for the PAH (see below).

### Coal Dust PAH Analysis

#### Gas Chromatography and Mass Spectrometry

Methylene chloride, hexane and DMSO extracts of 100 mg CD were analyzed using gas chromatography with mass spectrometry (GC/MS). Analysis was performed using an HP 5890 Series II GC with an HP 5972 Series MS as the detector. The GC/MS conditions are described in Table 1. All samples were run with blanks and in both scanning and selected ion monitoring (SIM) modes. The ions selected for monitoring were chosen based on a PAH cocktail QTM mixture from Supelco (#47930-U). The ions are the most discriminating ions for each PAH compound, and usually the most abundant.

**Table 1:** The concentrations of different PAHs in CD.

PAH	Concentration (μg/mg CD)	Estimated Exposure per CD-exposed rat^1^ (μg)
Phenanthrene	0.0300	1.200

Naphthalene	0.0139	0.556

Pyrene	0.0620	0.248

Fluoranthene	0.0580	0.232

Fluorene	0.0500	0.200

Total identifiable PAHs^2^	0.0605	2.436

^1^CD-exposed rats in this study received 40 mg CD by IT instillation. The micrograms of PAH instilled represents the estimate concentration in the 40 mg of CD.

^2^In addition to be identified PAHs, multiple methyl substituted naphthalenes and phenanthrenes were identified. Theses could not be quantified because of an absence of matching standards. Other PAHs and substituted PAHs maybe present but were either not detected or the presence of interfering ions prevented unambiguous identification.

#### HPLC DCM Sample Preparation

In addition, the methylene chloride extract of CD was analyzed using an HPLC clean-up method. The column used for this method was a Jordi Gel DVB 500 A 500 mm × 10 mm column supplied by Alltech (Cat # 100567). After contaminants were eluted off the column the PAHs can be eluted and collected using a six-port Valco valve. This fraction was then concentrated. The flow rate for the HPLC method is 1.5 ml/min and fractions are collected based on retention time and a fluorescent detector with an excitation wavelength of 254 nm and an emission wavelength of 400 nm. The HPLC system used was a GBC HPLC system consisting of the pump GBC LC1150, photodiode array GBC #LC5000, autosampler GBC #LC1650, inline degasser GBC #LC1460, and a Schimadzu fluorescence detector #RF-551. The software used to control the system and collect data was WinChrome v.1.32. For each run, 200 μl of sample was injected onto the column.

### Intratracheal Instillation

CD suspensions were made up daily from heat-sterilized samples using nonpyrogenic sterile 0.9% saline (Abbott Laboratories, North Chicago, IL). Suspensions were vortexed directly after preparation and shaken well before instillation. The CD particles were suspended in sterile saline at a concentration of 133.3 mg/ml as previously described [3]. Rats received a single intratracheal instillation of either 0.3 ml of this suspension (40 mg/rat) or 0.3 ml of saline (vehicle) by intratracheal (IT) instillation as previously described 14 days before necropsy [16-18]. Because of the black color of CD, its distribution to both left and right lung was verified at necropsy.

### Preparation of BNF

To prepare the BNF (Sigma Adrich Co., St. Louis, MO) suspension, the corn oil (Mazola) vehicle was sterilized by filtering with non-pyrogenic Acrodisc (Pall Gelman Sciences, Ann Arbor, MI) 25 mm syringe filter (0.2 μm in diameter). Solutions of 5% BNF (Sigma, St. Louis, MO) in sterilized corn oil (50 mg/ml) were prepared 24 h before injection. The suspension was vortexed and then sonicated for 15 minutes using an Ultronics sonicator (Mahwa, NJ). Rats received BNF daily for 3 days prior to euthanasia.

### Necropsy of Rats

Rats were euthanized by IP injection of sodium pentobarbital (Sleepaway^®^, Fort Dodge Animal Health, Fort Dodge, IA). The abdomen was opened by incision along the midline and the lungs and attached organs, including tracheobronchial lymph node, thymus, heart, aorta, and esophagus, were removed.

### Isolation of AT-II Cells

To assure that both normal and hypertrophied AT-II cells were isolated in this study, AT-II cells were isolated using a well-established method based upon cell attachment, rather than cell size [18]. In brief, this procedure is based upon the fact that elastase digestion of the lung predominantly yields cells with Fc receptors and AT-II cells, which do not have Fc receptors. The cells with Fc receptors are removed during initial plating on IgG-coated dishes [18]. This procedure produces a high yield of a purified AT-II cell population that is independent of cell size but is not considered quantitative.

### Evaluation of AT-II Cell Purity

The purity of AT-II cells was determined by staining the lamellar bodies with the lipophilic fluorescent Phosphine 3R dye (Roboz Surgical Instrument, Washington, DC) as previously described [19]. Briefly, the technique involves mixing 9 parts of cell suspension (1 × 10^7 ^cell/ml) with 1 part of 0.02% phosphine 3R dye (prepared by 10 mg dye dissolved in 10 ml PBS) followed by equilibration for 2 minutes. Forty μl of this mixture were spread over a slide and viewed under a fluorescent microscope using a FITC filter cube with 477 nm absorption and 512 nm emission. Six images were captured from each slide for counting AT-II cells. The percentage of AT-II cells from the total number of cells was calculated. In addition, since AT-II cell hypertrophy is a consequence of particle exposure [3,4], the cells were examined to assure that hypertrophied AT-II cells were recovered.

Fresh AT-II cell suspension (containing 1 million cells) was used for electron microscopy while the rest was fixed in freshly prepared 2% paraformaldehyde. Later on the same day, cytospin slides were prepared from the fixed cells using 50,000 cells/slide. Cytospin slides were stored in the refrigerator until stained.

### Detection of AT-II Cell Viability

The percentage viability of AT-II cells was determined by trypan blue exclusion as previously described [20].

### Electron Microscopy of AT-II Cells

Freshly prepared AT-II suspensions (containing 10^6 ^cells) were used for electron microscopic identification of AT-II cells as previously described [19]. Briefly, the cell pellets were preserved in Karnovsky's fixative, then postfixed at 4°C in 2% osmium tetroxide for 1 h, mordanted in tannic acid, and stained overnight at 4°C in 0.5% aqueous uranyl acetate. The samples were then dehydrated in alcohol and embedded in Epon. The sections were placed on 200-mesh grids and stained for 5 min with Reynold's lead citrate and for 20 min with 3% uranyl acetate. Photographs were taken on a Jeol 1220 transmission electron microscope at 80 kv. AT-II cells were identified by the presence of lamellar bodies.

### Quantitative Immunofluorescence for AhR in the Nuclei and Cytoplasm of Isolated AT-II Cells

Quantitative indirect immunofluorescence with co-localization is a quantitative procedure which allows the measurement of the amount of one fluorochrome that is within the same spacial location as a second fluorochrome using digital images [21]. In contrast to studies in isolated cells [22], in preliminary studies, AhR could not be demonstrated in intact lung tissue sections. Therefore, in this study, we isolated AT-II cells and used digital photomicroscopy, a specific nuclear stain (Cytox Green), and morphometric measurement to measure nuclear co-localization of AhR.

#### Immunofluorescent staining for AhR

Slides were stained using indirect immunofluorescence to detect and quantify AhR to determine its nuclear and cytoplasmic localization in isolated AT-II cells exposed to CD and then to PAH. Indirect immunofluorescence was conducted as previously described [22], with minor modification. Briefly, the area containing the cells on the cytospin slides was encircled by a hydrophobic marker. This allowed circumscribed application of antibodies to the marked area. Slides were then immersed in 2% paraformaldehyde to assure fixation before washing. Then slides were washed in distilled water followed by rinsing in PBS for 5 minutes. To avoid non-specific binding, slides were blocked by application of a few drops of freshly prepared filtered 1% bovine serum albumin (BSA) (Sigma Aldrich Co.).

A polyclonal rabbit anti-rat AhR antibody (BioMol Research Laboratories, Inc., Plymouth, PA) was diluted 1:20 with 1% BSA and 100 μl of the solution was applied on each cytospin slide for 1 h. For the negative control, the primary antibody was replaced by normal rabbit serum. Then 100 μl of an Alexa Fluor^® ^594 donkey anti-rabbit antibody (Molecular Probes, Eugene, OR) diluted 1:20 with PBS was applied on each cytospin slide for 1 h in the dark. Therefore, the antigen sites for AhR fluoresced red under a fluorescence microscope. The slides were then washed using distilled water and covered with anti-fade gel mount (Biomeda Corp., Foster City CA) before applying a cover slip.

#### Staining of AT-II Cell Nuclei

In order to identify nuclear AhR, the nucleus was stained with a green color using Cytox Green stain according to the manufacturer's direction (Molecular Probes, Inc., Eugene, OR).

#### Digital Photomicroscopy

For all immunofluorescence studies, five images were captured by a researcher blinded to the exposure status. The slides were first examined with an Olympus fluorescence photomicroscope (Olympus AX70; Olympus American Inc., Lake Success, NY) using two filters: (i) a green cube (460–500 nm excitation) and (ii) a red cube (532.5–587.5 nm excitation). Criteria for specific staining were: (i) absence of staining in the negative controls, (ii) absence of background staining, and (iii) distinct cellular localization of AhR. Then, photomicrographs were acquired for quantitative morphometric analysis using a 40× objective and a Quantix cooled digital camera (Quantix Photometrics; Tucson, AZ) with QED camera plugin software (QED Imaging, Inc., Pittsburgh, PA). The digital camera settings for contrast, brightness and gamma were held constant during the capture time of all images.

#### Morphometric measurement of immunofluorescence

Morphometric measurements were made using commercial morphometry software (Metamorph Universal Image Corp., Downingtown, PA). By using this software, the area of fluorescence for AhR in the cytoplasm and in the nucleus of AT-II cells was measured and expressed as μm^2^. The most important parameters deduced by the aid of morphometric analysis included the following:

1) The proportional nuclear AhR expression is the area of AhR expression in the nucleus relative to the total AT-II cell nuclear area (areas where Cytox green is present). This measurement is calculated from the following formula:



where,

P is the proportional AhR localization in the nucleus (Cytox green).

R is the percentage of AhR colocalized within the Cytox green nuclear matrix.

T is the total AhR area (in the nucleus and cytosol).

G is the total nuclear area (Cytox green).

2) The proportional cytosolic AhR expression is the area of AhR expression in the cytoplasm relative to the total nuclear area (areas where Cytox green is present). This measurement is calculated from the following formula:



where,

L is the proportional AhR localization in cytoplasm determined by the equation.

M is the percentage of AhR area not colocalized with the cytox green nuclear marker.

T is the total AhR area (in the nucleus and cytosol).

G is the total nuclear area (Cytox green) determined by Metamorph software.

This measurement is important because it determines the amount of AhR remaining in the cytoplasm after induction by the CYP1A1 inducer, BNF.

3) The percentage of AT-II cells with AhR localized in the nucleus was manually calculated from distinct images. The AT-II cells with AhR localized in their nucleus and the total number of AT-II cells was counted in 5 images per rat. The cells expressing AhR in the nucleus have a yellow color due to colocalization of green and red fluorochromes.

4) The total area of AhR expression per AT-II cell. The total expression area (in nucleus and cytoplasm) of AhR was measured as micrometers squared and was divided by the number of AT-II cells.

### Statistical Analyses

All analyses were performed with SAS/STAT software, Version 8.2 utilizing the Proc Mixed procedure. Dependent variables were analyzed using two-factor analysis of variance using two-factor mixed model analysis of variance (CD/Saline by Oil/BNF). All pairwise comparisons where appropriate were performed with Fisher's LSD. All results were considered statistically significant at P < 0.05.

## Results

### Coal Dust PAH Analysis Results

Several PAHs were identified in the CD including phenanthrene, naphthalene, pyrene, fluoranthene and fluorine (Table 1). The concentrations of PAHs identified in 100 mg CD are described in Table 1. From that table, the amount of different types of PAHs was calculated per 40 mg CD, which is the dose used for IT instillation. The total amount of PAHs calculated per CD-exposed rat was 2.436 μg. Since BNF-treated rats received 50 mg/kg of BNF, the dose of BNF is 1250 μg for rats with an average weight of 250 g. Therefore, the dose of BNF was more than 500 times the total PAHs quantified in CD.

### The Number and Purity of Isolated AT-II Cells

**Figure 1:**
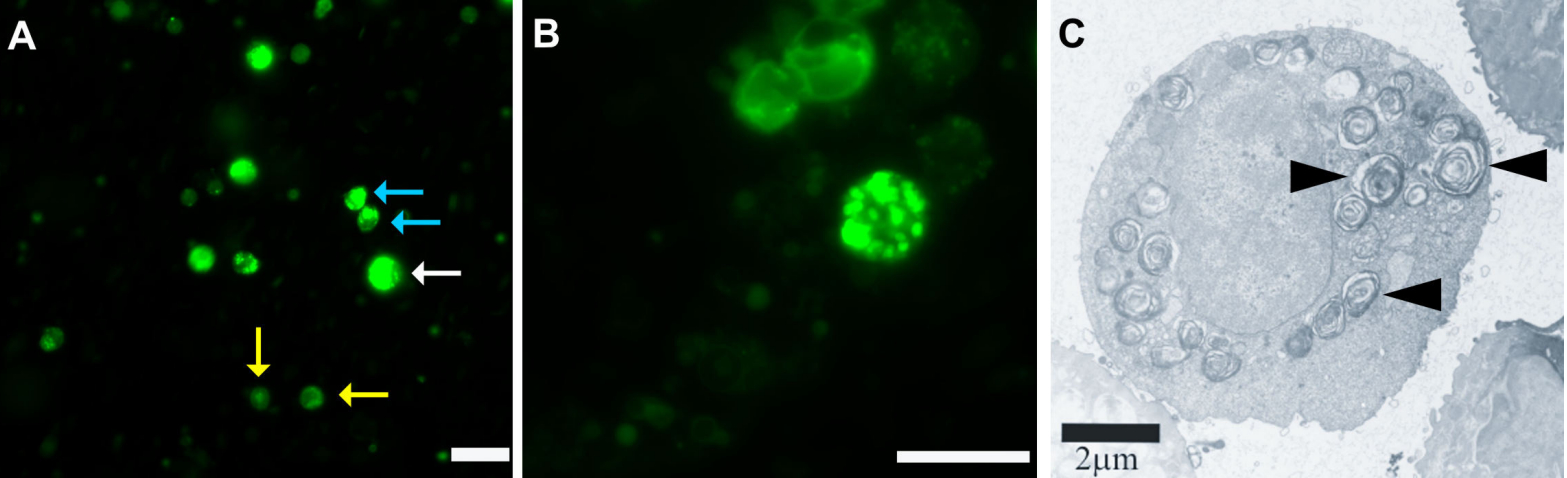
**Identification of isolated AT-II cells with phosphine 3 R fluorescent dye**. (A) A representative image from CD-exposed rat showing the staining of AT-II cell with bright green color. Note the presence of a hypertrophied AT-II cell (white arrow) that is larger in size than the other cells (blue arrows). Cells that are not AT-II cells (yellow arrow) do not get the stain. Reference bar is 20 μm. (B) An image of an AT-II cell captured under higher magnification demonstrating the lamellar bodies and the cells close to it do not have lamellar bodies and were classified as non AT-II cells. Reference bar is 20 μm. (C) A representative electron microscope image of an AT-II cell showing the lamellar bodies (black arrow heads). Reference bar is 2 μm.

AT-II cells stained with phosphine 3R contained bright green lamellar bodies that are absent in other pulmonary cell types (Figure 1A and 1B). Hypertrophied alveolar type II cells were recovered from the coal dust-exposed but not control rats using this isolation procedure (Figure 1A).

**Figure 2:**
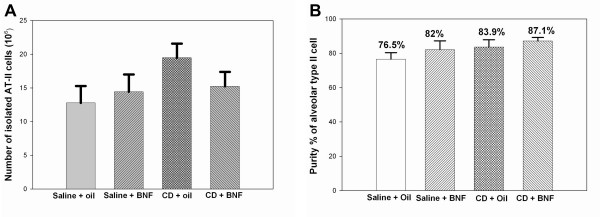
**The number (A) and purity (B) of AT-II cells in different treatment groups are shown**. No significant changes in the number or purity of isolated type II cells among groups were observed. Results are means ± SE, n = 4 in all groups except in saline plus oil group (n = 3).

Using transmission electron microscopy, lamellar bodies were also visualized within AT-II cells (Figure 1C). The number of AT-II cells obtained by isolation and counted by a Coulter multisizer ranged from 12.8 – 19.9 million/rat and was numerically higher but not significantly increased by exposure (Figure 2A). The purity ranged from 76.5 – 80.1% (Figure 2B) and was unaffected by exposure. Because the isolation method does not recover all alveolar type II cells from the rat lung, no attempt was made to quantify the numbers of enlarged type II cells.

### General Morphometric Findings

**Figure 3:**
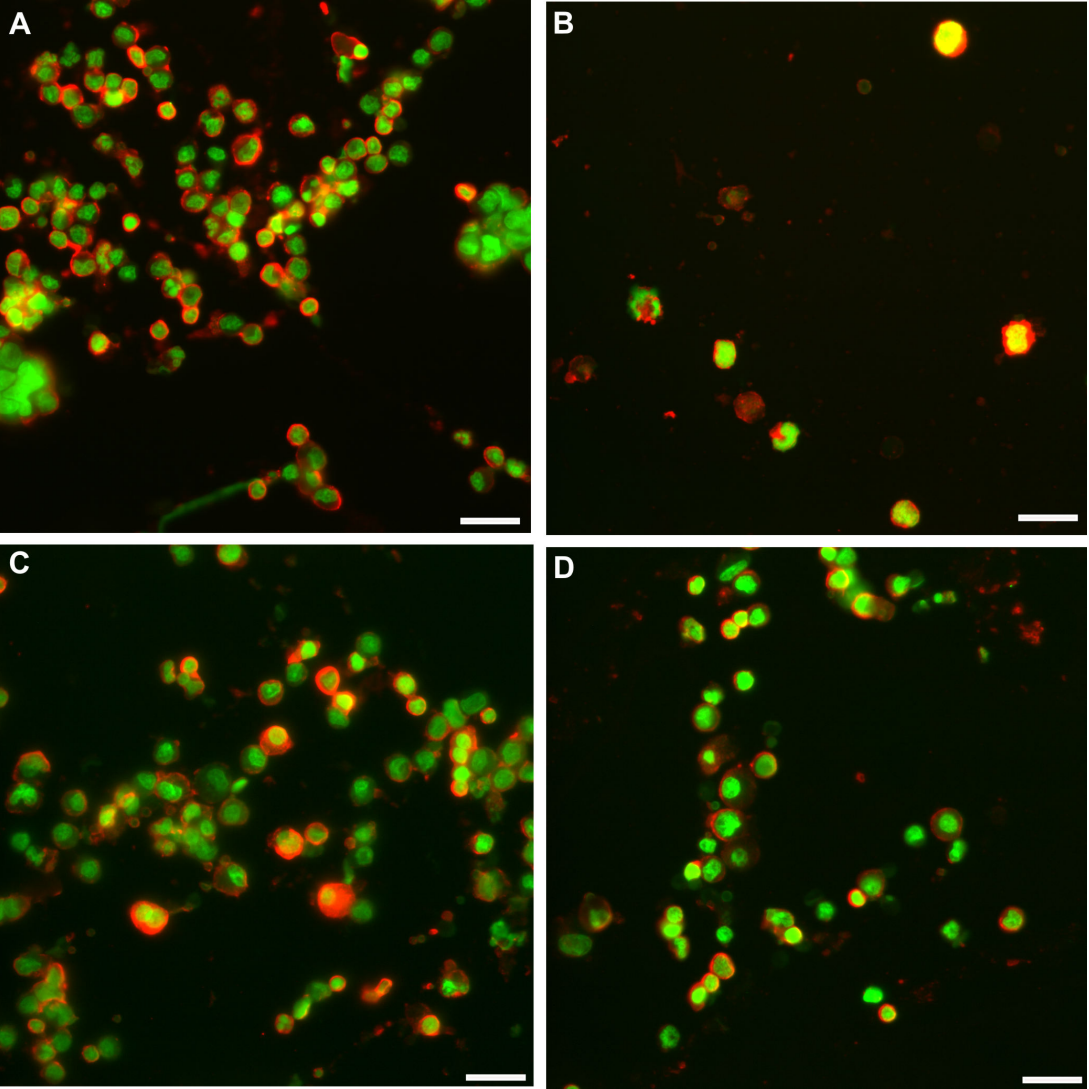
**Representative immunofluorescent images showing the expression of AhR in AT-II cells in rats exposed to saline or CD with and without BNF**. In A, B, C and D the AhR stained red, the nuclear area stained green, and the overlapping of the green area by red area produces yellow color representing localization of AhR in the nucleus. (A) An image of AhR and Cytox green from saline plus oil group. (B) An image of AhR and Cytox green from saline plus BNF group showing distinct localization of AhR in AT-II cell nucleus (yellow color). (C) An image of AhR and Cytox green from CD plus oil group. (D) An image of AhR and Cytox green from CD plus BNF group showing localization of AhR in AT-II cell nucleus (yellow color). Reference bar is 20 μm.

The fluorochrome used for detection of AhR was Alexa Fluor^® ^594 (Molecular Probes, Eugene, OR), which fluoresced red at the sites of expression. Cytox green produced green nuclear fluorescence. Therefore, nuclear expression of AhR produced yellow fluorescence (Figure 3).

#### Localization of AhR in the AT-II Cell Nucleus

**Figure 4:**
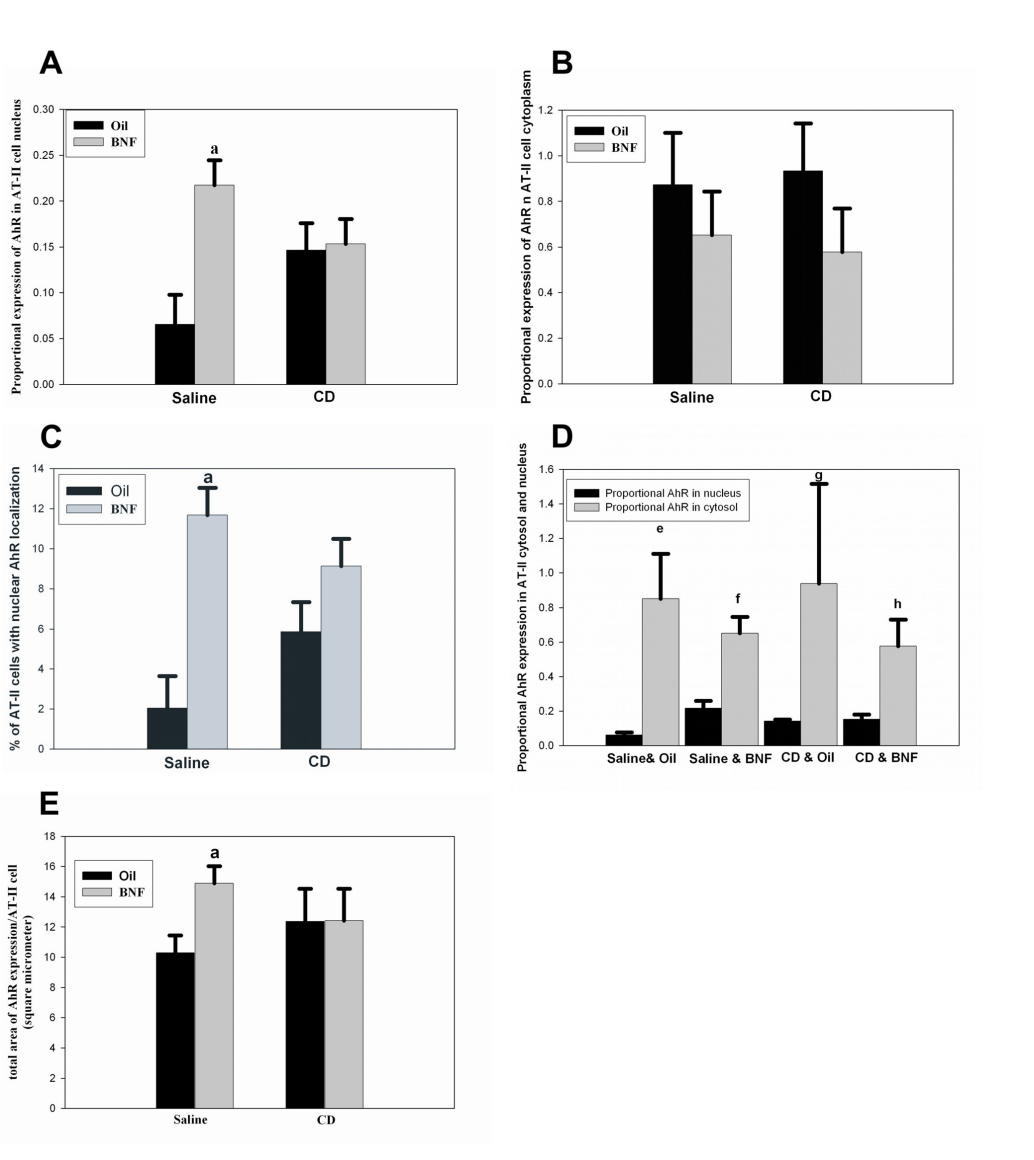
**Morphometric quantification of AhR in the nucleus and cytoplasm of isolated AT-II cells**. (*A*) The proportional AhR in the nucleus was significantly increased (letter a; *p *< 0.05) in the saline plus BNF group compared to the saline plus oil group but no increase in nuclear AhR was observed after BNF in co-treated rats. (*B*) No significant change in the proportional AhR expression in cytoplasm of rats receiving BNF compared to those receiving oil was observed in either the IT saline or CD groups. (*C*) The percentage of AT-II cells with AhR localized in the nucleus was significantly increased (letter a; *p *< 0.05) in BNF-exposed rats without CD exposure but not in BNF and CD exposed rats. (*D*) The proportional AhR expression in AT-II cell cytosol was significantly higher (*P *< 0.05) than that in the nucleus in all treatment groups (letters e, f, g, and h, respectively). (*E*) The total area of AhR expression per AT-II cell was significantly increased (*p *< 0.05) in rats receiving saline plus BNF compared to those receiving saline plus oil (letter a; *p *< 0.05) but BNF causes no changes in rats receiving CD. Results are mean + SE, n = 4 in all groups except in saline plus oil group (n = 3).

BNF caused an increase in proportional AhR nuclear localization in rats without CD exposure (*P *= 0.027). In rats exposed to CD, the proportional AhR nuclear localization was not significantly affected by BNF compared to exposure to CD alone (Figures 3C, D and Figure 4A).

#### Localization of AhR in AT-II Cell Cytosol

The proportional AhR expression in AT-II cell cytosol showed non-significant reductions in rats receiving BNF compared to rats without BNF exposure in both the saline and CD groups (Figure 4B).

#### Percentage of AT-II Cells with nuclear localization of AhR

In rats that received IT saline instead of CD, the percentage of AT-II cells with AhR localized in the nucleus was significantly increased by BNF (P = 0.0008). However, in rats that received IT CD, the percentage of AT-II cells with AhR localized in the nucleus was not significantly affected by BNF (Figure 4C).

#### Proportional AhR Expression in Cytosol versus Nucleus

By morphometric analysis, the proportional area of AhR in the cytoplasm was greater than the proportional area in the nucleus in all treatment groups (Figures 4D) (P < 0.05).

#### Total area of AhR expression per AT-II cell

In rats that received IT saline, the total expression area (in nucleus and cytoplasm) of AhR (measured as square micrometer per AT-II cell) was significantly increased (P < 0.036) by BNF. In rats that received IT CD, this area was not significantly changed by BNF exposure (Figure 4E).

## Discussion

Previous data from our laboratory showed that exposure to foreign particles, such as CD and silica suppresses the PAH-mediated induction of CYP1A1 in rat lung [3,4,23]. By histopathologic assessment, particle exposure produces AT-II cell hyperplasia and hypertrophy in the lungs of rats exposed to either respirable silica or respirable coal dust [3,4,23]. Previous studies indicated that AT-II cell hypertrophy and hyperplasia were related processes and a response to alveolar type I cell injury in the particle-exposed lung [24]. Using immunofluorescent co-localization of CYP1A1 and the AT-II cell markers cytokeratins 8/18 in CD-exposed rats, studies from our laboratory showed that these hyperplastic and hypertrophic cells expressed decreased amounts of PAH-induced CYP1A1 protein. This suggested that CD exposure resulted in appearance of a new population of AT-II cells with diminished capacity for CYP1A1 induction [3,4]. Since CYP1A1 is induced by ligand activation of the AhR, which is localized in the cytoplasm in the inactive state, we isolated AT-II cells from *in vivo *CD-exposed rats to localize and quantify AhR in the cytoplasm and nucleus after *in vivo *treatment with the specific CYP1A1 inducer, BNF.

AhR translocation is essential for CYP1A1 induction by PAHs. After being stimulated by an inducer, the AhR is translocated to nucleus and binds to another protein called AhR nuclear translocator (ARNT) forming a heterodimeric protein complex [25]. This complex binds to the xenobiotic responsive element (XRE) located at the enhancer region of CYP1A1 gene producing conformational changes in chromatin structure and initiates CYP1A1 transcription [26].

Morphometric analysis of immunofluorescence staining in this study showed that the proportional AhR expression in AT-II cell nuclei, an indicator of AhR localization in the nucleus, was significantly increased in BNF-exposed rats compared with vehicle control rats. Moreover, the total area of AhR expression per AT-II cell showed a significant increase in BNF-exposed rats compared to vehicle controls. This finding is consistent with many studies involving BNF as a specific potent inducer for CYP1A1 through activation of AhR and served as a positive control for the immunofluorescent procedure used in our study [27-32].

Conversely, in CD exposed rats, the localization of AhR in AT-II cell nuclei, measured as a proportional AhR expression in the cell nucleus, was not significantly affected by BNF. In addition, in CD-exposed rats, the percentage of AT-II cells with nuclear AhR expression was not significantly affected by BNF. These findings suggest that the mechanisms of CD-induced inhibition of CYP1A1 induction may involve failure of the CD-exposed cells to translocate AhR after PAH exposure. One possible explanation for the alteration of AhR localization in AT-II cell nucleus of CD-exposed rats after being activated by BNF is AhR proteolysis. Since CD contains small amounts of PAHs and ligand-activated AhR is rapidly proteolyzed resulting in an immediate reduction of the AhR number [33-36], it is possible that the prior exposure to low levels of PAH decreased the AhR pool in the cell. Gu and co-workers suggested that this ligand-dependant degradation of AhR aims to ameliorate the response of the cell to environmental changes, thereby protecting the cell from prolonged exposure to excessive concentrations of agonists as an adaptive mechanism [33]. However, the amount of PAHs quantified in CD was 500 times less than the dose of BNF injected. Furthermore, the BNF used in this study was injected daily for three days to repeatedly activate AhR. Thus, increased AhR proteolysis due to prior PAH-activation in the CD-exposed rats seems unlikely.

The decreased AhR nuclear translocation in alveolar type II cells after CD exposure may well be a general response of alveolar type II cells to inflammation associated with lung-deposited particles, since CYP1A1 induction and its dependent activity (EROD) were inhibited by exposure to another type of occupational dust, crystalline silica, which does not contain PAH [23]. Histopathological and bronchoalveolar lavage examinations showed that both CD and silica were associated with pulmonary inflammation [3,23,37-39]. Inflammation can downregulate CYP-dependent activity in the liver [40-42]. In the liver, a number of different CYPs are suppressed by inflammation and reduced CYP activity involves both transcriptional and post-transcriptional mechanisms [43]. Because exposure to CD is associated with pulmonary inflammation [3], the mechanism of suppressed PAH-induced CYP1A1 activity in the particle-exposed lung is likely to involve proinflammatory mediators known to influence CYP1A1 activity. In particular, *in vitro *NF-κB suppresses PAH-induced CYP1A1 activity by preventing acetylation of the CYP1A1 promoter and thereby reducing transcription [8,44,45]. In addition, nitric oxide binds to the catalytic heme moiety of CYP1A1, leading to post-transcriptional downregulation of CYP1A1 activity [46]. Indeed, our previous studies showed, that PAH induced CYP1A1 activity was inversely related to CD-induced pulmonary inflammation [3].

However, in both the silica and coal dust-exposed rat lungs, alveolar type II cells proliferate following pulmonary inflammation and are often refractory to CYP1A1 induction [3,23]. A previous *in vitro *study with cells of apparent alveolar type II cell origin indicated that proliferating cells had less constituitive EROD activity than confluent (quiescent) cultures [47]. In a keratinocyte cell line, differentiated cultures had more AhR mRNA and more TCDD-inducible CYP1A1 than did proliferating cultures [48]. Similarly, *in vivo *rat skin cells that remain refractory to CYP1A1 induction are predominantly basal cells, the progenitor population of the skin [49]. In this study, we have investigated the hypothesis that like proliferating keratinocytes, alveolar type II cells from CD-exposed rats have decreased AhR translocation to the nucleus following *in vivo *PAH exposure. Our findings are consistent with these previous findings for other proliferating cells and indicate that the alveolar type II cells of the CD-exposed rat lungs have decreased capacity for BNF-induced AhR nuclear translocation. Thus, suppression of PAH-induced CYP1A1 activity in the CD-exposed lung is associated with both inflammation and the expansion of an alveolar type II cell population with reduced capacity for BNF-induced AhR nuclear translocation.

Since CYP1A1 induction is dependent upon AhR nuclear translocation [9,50], the alveolar type II cells of the particle-exposed lung would be expected to produce fewer CYP1A1-dependent metabolites of PAH carcinogens. It is these PAH metabolites which are believed to cause the DNA adducts which may initiate lung cancer and AT-II cells are potential progenitor cells for lung cancer [51,52]. Thus, our findings indicate CD exposure prior to PAH exposure decreases the inducibility of PAH metabolism in the lung, at least in part because there is diminished capacity for AhR nuclear translocation in the type II cells. However, if DNA adducts have already caused DNA mutations in alveolar type II cells, expansion of initiated cells is integral to the process of cancer promotion [53,54]. Studies are still needed to determine the effect of CD upon the lung that has been previously exposed to PAH carcinogens. It may well be that CD is actually a complex modifier of PAH carcinogenesis with both the negative modification of PAH-induced metabolism indicated in this and previous studies [3,4,23] and the capacity for promotion through expansion of initiated cells.

Taken together, our results demonstrate that exposure of rats to CD modifies nuclear translocation of AhR in AT-II cells after subsequent BNF exposure. This provides an explanation for at least some of the diminished CYP1A1 induction observed in the particle-exposed lung upon subsequent BNF exposure.

## Abbreviations

AIP: AhR-interacting protein; AT-II: alveolar type II; AhR: aryl hydrocarbon receptor; AHRE1 and AhRE2: AhR responsive elements1 and 2; Arnt: AhR-nuclear translocator; Bax: BCL2-associated X protein; BNF: beta-naphthoflavone; CD: coal dust; CNSPE: cyanopropyl solid phase extraction; CYP1A1: cytochrome P4501A1; GC/MS: gas chromatography with mass spectrometry; HSP90: heat shock protein 90; IgG;: immunoglobulin-G; EROD: 7-ethoxyresorufin-*O*-deethylase; DCM: methylene chloride; PAH: polycyclic aromatic hydrocarbon; (SIM): selected ion monitoring; TCDD: 2, 3, 7, 8, – tetrachlorodibenzo- p-dioxin; XRE: xenobiotic responsive element.

## Disclaimer

The findings and conclusions in this report are those of the author(s) and do not necessarily represent the views of the National Institute for Occupational Safety and Health.

## Competing interests

The authors declare that they have no competing interests.

## Authors' contributions

MG participated in the study design, conducted the experiments, and drafted the manuscript. LB planned many of the experiments, participated in the conduct of the experiments, and assisted in the manuscript preparation. BL planned, conducted, and wrote up the coal dust PAH analysis. VC and JN contributed to the experimental design, acquisition of funding and the writing of the manuscript. MK participated in the study design and the statistical analysis. AH conceived of the study, participated in its design, acquired the funding, supervised the conduct of the experiments and helped in the drafting the manuscript.
